# *Gossypium hirsutum* gene of unknown function, Gohir.A02G044702.1, encodes a potential B3 Transcription Factor of the REM subfamily

**DOI:** 10.17912/micropub.biology.000574

**Published:** 2022-08-06

**Authors:** Michael Allen, Amanda M. Hulse-Kemp, Amanda R. Storm

**Affiliations:** 1 Department of Biology, Western Carolina University, Cullowhee, NC; 2 Genomics and Bioinformatics Research Unit, The Agricultural Research Service of U.S. Department of Agriculture, Raleigh, NC; 3 Department of Crop and Soil Sciences, North Carolina State University, Raleigh, NC

## Abstract

A gene of unknown function, Gohir.A02G044702.1, identified in *Gossypium hirsutum* was studied using sequence and structure bioinformatic tools. The encoded protein (UniProt A0A1U8MGX4) was predicted to localize to the nucleus, was found to retain the B3 transcription factor domain with conserved DNA-binding residues and to most closely cluster with REM subfamily members of B3-domain containing proteins.

**Figure 1. Sequence and Structure Characterization of GhB3-A0A1U8MGX4:**
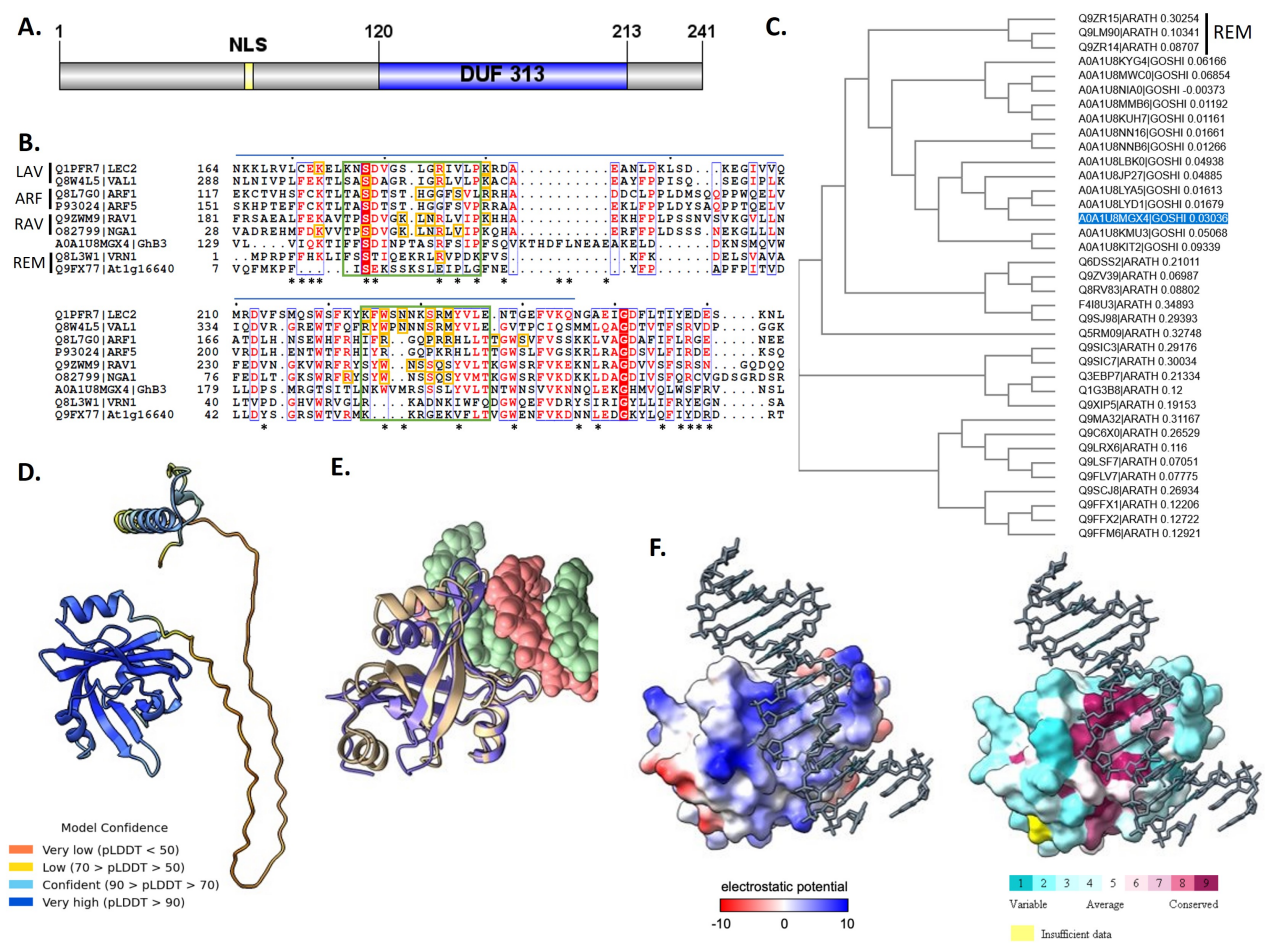
(A) Domain architecture of GhB3-A0A1U8MGX4 indicating the location of predicted sequence features: Created using IBS (Wenzhong et al., 2015) based on predictions from InterPro (Blum et al., 2020), BUCSA (Savojardo et al., 2018), and LOCALIZER (Sperschneider et al., 2017), NLS - nuclear localization sequence, DUF 313 domain (PF03754). (B) Multi-Sequence Alignment of DUF 313 domain region of GhB3-A0A1U8MGX4 and B3 domain-containing proteins with known structures from each subfamily : LAV (LEC2 - PDB 6J9C, VAL1 - PDB 6J9A, Tao et al., 2019), ARF (ARF1 - PDB 4LDX, ARF5 - PDB 4LDU, Boer et al., 2014), RAV (RAV1 - PDB 1WID, Yamasaki et al., 2004; NGA1 - PDB 5OS9, Sasnauskas et al., 2018), REM (VRN1 - PDB 4I1K, Gordon et al., 2013; At1g16640 - PDB 1YEL, Waltner et al., 2005), Created with ClustalOmega (Madeira et al., 2019) and ESPript3 (Robert and Gouet, 2014) and annotated to show location of the DUF 313 domain (blue bar), ConSurf-identified highly conserved sequences in GhB3-A0A1U8MGX4 (*), DNA-interacting N- and C-arm loops (green boxes) and residues implicated in DNA binding either by crystal structures or mutagenesis (yellow boxes). (C) Phylogenetic tree of cotton ( *Gossypium hirsutum* ) and *Arabidopsis thaliana* GhB3-A0A1U8MGX4 homologs: homologs identified using PhyloGenes (Zhang et al., 2020) and tree created using ClustalOmega, the GhB3-A0A1U8MGX4 sequence is highlighted (blue) and the 3 Arabidopsis homologs identified as belonging to the REM subfamily are indicated. (D) AlphaFold model structure for GhB3-A0A1U8MGX4 with coloration based on model confidence. (E) Structure overlay of GhB3-A0A1U8MGX4 model (beige ribbon) and LEC2-DNA complex (purple ribbon and space-filling DNA, PDB 6J9C). (F) GhB3-A0A1U8MGX4 model with DNA showing (left) electrostatic surface calculated by ChimeraX and (right) surface coloring based on ConSurf conservation scores.

## Description

Introduction

In a recent sequencing of the genome of upland cotton, *Gossypium hirsutum* (L. accession Texas Marker-1 (TM-1) version 2.0 and annotation version 2.1) (Chen et al., 2020), the gene, LOC107936594 (CottonGen: Gohir.A02G044702_UTX-TM1_v2.1), was identified to encode a conserved protein of unknown function labeled 'B3 domain-containing protein At1g05920-like' (Gohir.A02G044702.1; NCBI: XP_016724843; UniProt: A0A1U8MGX4). Here we present evidence that supports this protein, referred to here as GhB3-A0A1U8MGX4, is part of the B3 domain-containing family, likely within the highly diverse REM subfamily, and contains the sequence and structure features required for nuclear-localization and DNA-binding to function as a transcription factor (TF). Members of the B3 superfamily all contain a B3 domain, a ~100 amino acid DNA-binding domain specific to plants and found in even unicellular green algae (Swaminathan, Peterson, Jack, 2008). It is prevalent in flowering plants with around 100 B3-containing proteins identified in Arabidopsis (Wang et al., 2012). This superfamily is involved in regulating many key processes, including stress and hormone responses (Yamasaki, 2016; Gordon et al., 2013; Boer et al., 2014), embryogenesis (Tao et al., 2019) and development (Waltner et al., 2005). The superfamily has been classified phylogenetically into four subfamilies: LAV (LEC/ABI/VAL), RAV (Related to ABI3/VP1), ARF (Auxin Response Factor) and REM (Reproductive Meristem), which differ in their DNA recognition sequence (Romanel et al., 2009).

Sequence Features

InterPro webserver identified the 241–amino acid GhB3-A0A1U8MGX4 protein as a member of the ‘DNA-binding pseudobarrel domain’ superfamily (IPR015300) and the ‘B3 domain-containing protein At2g31720-like’ family (IPR005508) with a domain of unknown function DUF 313 (PF03754). This plant-specific family of unknown function includes At2g31720, Auxin Response Factor 70 (ARF70, UniProt Q8RV83), a protein linked to stress response through yeast one-hybrid screening of TF-promoter interactions (Ikeuchi et al., 2018). Sequence analysis of GhB3-A0A1U8MGX4 by subcellular localization programs BUSCA and LOCALIZER predicted a nuclear localization sequence (NLS), KRKR, in the N-terminal region, in agreement with the function as a transcription factor. A domain architecture was created to visualize these sequence features ( **Figure 1A** ). ConSurf (Ashkenazy et al., 2016) was used to calculate the evolutionary conservation of each residue and the most highly conserved residues were found within the DUF 313 domain region (amino acids 120-213), these residues are indicated in the multi-sequence alignment by asterisks ( **Figure 1B** ). The full ConSurf results are available as Extended Data.

Homology

Of the 40 plant and 10 non-plant genomes used by the PhyloGenes webserver, homologs of GhB3-A0A1U8MGX4 were found in 25 species, confined to magnoliophyta (angiosperms), which agrees with the species listed by pfam for DUF 313 domain (PF03754) containing proteins. Arabidopsis ARF70 (Q8RV83) is a listed homolog but none of the other proteins have been functionally characterized or named. This indicates that these proteins belong to a set of B3 domain-containing proteins distinct from any currently characterized member of this family. PhyloGenes identified 14 cotton paralogs and 23 Arabidopsis orthologs of GhB3-A0A1U8MGX4. ClustalOmega was used to create a multi-sequence alignment (available as Extended Data) and a phylogenetic tree (Neighbor-joining) of these sequences ( **Figure 1C** ). The cotton paralogs separate into two distinct clusters with the most closely aligned Arabidopsis sequences to GhB3-A0A1U8MGX4 (Q9ZR15, Q9LM90, Q9ZR14) being proteins of unknown function but listed as belonging to the REM subfamily of B3 domain-containing proteins (Wang et al., 2012).

Structure Features

The AlphaFold tool in UCSF ChimeraX (Version 1.3, Pettersen et al., 2021; Jumper et al., 2021) was used to predict a structure model (available as Extended Data) for the GhB3-A0A1U8MGX4 protein ( **Figure 1D** ). The model showed a high level of confidence across amino acids 111-241, which includes the entirety of the DUF 313 domain region. This domain showed a seven-stranded, beta-sheet open barrel flanked by two short alpha helices characteristic of B3 DNA binding domains (Yamasaki et al., 2016). The lower confidence N-terminal 110 amino acids were deleted from the structure for remaining structure analyses.

A structure analog search using the DALI server (Holm, 2020) identified structures of B3 domain-containing proteins from each of the four subfamilies (LAV, RAV, ARF and REM) that closely matched the GhB3-A0A1U8MGX4 model (Z-scores between 9.7-11.1 and r.m.s.d values of 2.3-3.1 across 90-109 residues) even though sequence similarity was low (10-27%). Two structures were chosen from each subfamily and their sequences were aligned with GhB3-A0A1U8MGX4 using ClustalOmega and ESPript3 to create a multi-sequence alignment ( **Figure 1B** ). The multi-sequence alignment (MSA) shows the domain region where the greatest similarity was seen. The GhB3-A0A1U8MGX4 sequence retains many of the residues conserved across subfamilies (K134, S139, D140, D181, V212, G220) along with a number of residues identified as involved in DNA binding in other homologs (yellow boxes) which ConSurf also identified as evolutionarily conserved (asterisks).

The aligned sequences separated based on their known subfamilies with the GhB3-A0A1U8MGX4 sequence clustering most closely with the REM representative sequences (VRN1 and At1g16640). Subfamilies have been reported to have characteristic motifs within the DNA-interacting C-arm loop (RAV: 'WN/RSSQS'; ARF: 'RGQPK/RR'; LAV: 'WPNNKSR'; REM: deletion of 3-4 residues) (Swaminathan, Peterson, Jack, 2008). These motifs can be seen for each subfamily within the MSA but it is interesting that the GhB3-A0A1U8MGX4 C-arm loop sequence doesn’t match any of these motifs. Differences in this region have been attributed to DNA-binding specificity so this could indicate that GhB3-A0A1U8MGX4 binds to a distinct DNA sequence. This is still in agreement with belonging to the REM subfamily as it is known to be the largest and most diverse subfamily with highly diverged DNA binding (Romanel et al., 2009).

DNA was modeled into the predicted binding site using a structure overlay between the GhB3-A0A1U8MGX4 model and a LEC2-DNA structure (PDB 6J9C) using UCSF ChimeraX MatchMaker ( **Figure 1E** ). There is good alignment (r.m.s.d. of 1.014 angstroms over 53 of 96 pruned atom pairs) with DNA-interacting N- and C-arm loops associating with the major groove. A characteristic feature of B3 DNA-binding domains is a large positively charged area around the DNA binding site (Yamasaki et al., 2004). The GhB3-A0A1U8MGX4 model contains a similar distinct and large area of positively charged residues in the modeled binding site. Mapping of ConSurf conservation scores onto the structure indicates that these residues are also highly conserved ( **Figure 1F** ).

Conclusion

Evidence from the sequence, homology and structure analyses support GhB3-A0A1U8MGX4 being a B3 domain-containing protein within the REM subfamily, with a retained DNA-binding site. The nuclear subcellular localization and conserved and charged binding site features indicate a DNA binding function such as a transcription factor, similar to other members of this family, although potentially with a unique DNA recognition sequence. With a distinct binding motif and largely uncharacterized homologs, this protein could belong to a new subset of REM proteins involved in regulating a distinct plant process, likely specific to angiosperm species.

## Extended Data

Description: ConSurf sequence conservation results for A0A1U8MGX4 . Resource Type: Dataset. DOI: 10.22002/D1.20239

Description: AlphaFold modeled structure of A0A1U8MGX4. Resource Type: Model. DOI: 10.22002/D1.20240

Description: ClustalOmega MSA of PhyloGenes phylogenetic tree. Resource Type: Dataset. DOI: 10.22002/D1.20241
